# Complete Genome Sequence of *Brevibacterium linens* SMQ-1335

**DOI:** 10.1128/genomeA.01242-16

**Published:** 2016-11-10

**Authors:** Alessandra G. de Melo, Simon J. Labrie, Jeannot Dumaresq, Richard J. Roberts, Denise M. Tremblay, Sylvain Moineau

**Affiliations:** a Département de Biochimie, de Microbiologie et de Bio-informatique, Faculté des Sciences et de Génie, Québec, Québec, Canada; b Groupe de Recherche en Écologie Buccale, Faculté de Médecine Dentaire, Université Laval, Québec, Québec, Canada; c Département de Microbiologie et d’Infectiologie, Centre Hospitalier Afﬁlié Universitaire Hôtel-Dieu de Lévis, Lévis, Québec, Canada; d New England Biolabs, Ipswich, Massachusetts, USA; e Félix d’Hérelle Reference Center for Bacterial Viruses, Faculté de Médecine Dentaire, Université Laval, Québec, Québec, Canada

## Abstract

*Brevibacterium linens* is one of the main bacteria found in the smear of surface-ripened cheeses. The genome of the industrial strain SMQ-1335 was sequenced using PacBio. It has 4,209,935 bp, a 62.6% G+C content, 3,848 open reading frames, and 61 structural RNAs. A new type I restriction-modification system was identified.

## GENOME ANNOUNCEMENT

*Brevibacterium linens* is a Gram-positive bacterium found on the surface of a variety of washed rind cheeses produced globally (1–3). This non-spore-forming, halotolerant, strictly aerobic chemoorganotroph undergoes a rod-cocci cycle during growth and possesses mesodiaminopimelic acid in its cell wall (1, 2, 4). *B. linens* plays key roles during cheese ripening in the breakdown of lipids and proteins (2), the production of volatile sulfur compounds (5, 6), and the development of color due to carotenoid pigment production (1, 2, 4, 7). Furthermore, the production and accumulation of compatible solutes allows *B. linens* to grow in hyperosmotic environments (4). The genomes of three *B. linens* strains are currently available, of which one is complete (GCA_001606005.1) and two are scaffold and draft sequences (GCA_000167575.1 and GCA_000807915.1, respectively).

The genome of *B. linens* SMQ-1335 was sequenced using one SMRT cell in a PacBio RSII sequencer (Génome Québec Innovation Centre, Montréal, QC, Canada), which generated 76,075 raw subreads of an average length of 9,010 bp that provided an average coverage of 162-fold. The genome was assembled into one contig using HGAP (8). BLASR (9) was used to align and preassemble the sequences using the longest reads as seeds to which all the other subreads were recruited and mapped to correct random errors. The Celera assembler (10) was then used to *de novo* assemble these long and corrected reads into contigs. The sequences were refined using Quiver, wherein the raw reads were aligned on the contigs to generate a consensus sequence containing the complete genome. The single contig had redundant ends of 16,018 bp that were removed from one end for the final assembly. The origin of the genome was set upstream of the gene coding for the replication initiator protein DnaA. Gene prediction and annotation was performed using RAST (11) and BLASTp (12). The *B. linens* SMQ-1335 genome has a high G+C content (62.6%) and is composed of 4,209,935 bp, 3,848 genes, and 61 structural RNAs (49 tRNAs and 12 rRNAs).

*In vitro* tests revealed that *B. linens* SMQ-1335 is sensitive to vancomycin, daptomycin, gentamicin, tetracycline, and rifampin. However, this strain was insensitive to β-lactam antibiotics (penicillin and ceftriaxone), trimethoprim-sulfamethoxazole (TMP-SMX), and a second-generation fluoroquinolone (ciprofloxacin). A gene likely coding for the lantibiotic linocin was identified using BAGEL3 (13).

No genes coding for known toxins were found in the genome of SMQ-1335 using Virulence Finder (14), Virulence Factor Database (15), and DBETH (16). RAST and PHASTER (17) identified a putative prophage (31,300 bp). Analysis of this prophage sequence revealed many transposases and integrases, suggesting that this may be a nonfunctional prophage.

The methylome (18) of *B. linens* SMQ-1335 was analyzed to identify DNA methyltransferases and restriction endonucleases (19) as well as specificity subunits (20). The new methyltransferase M.Bli1335I, the restriction endonuclease Bli1335IP, and a new type I RM system were assigned, with the recognition site sequence CGGANNNNNNTTC. The SMQ-1335 genome may contain additional RM systems (types II, III, and IV). For the type II, a new restriction endonuclease (Bli1335II) was also assigned with DTGAAT as the recognition sequence.

### Accession number(s).

The complete genome sequence of *B. linens* SMQ-1335 is available in GenBank under the accession number CP017150.

## References

[1] Motta AS, Brandelli A 2008 Properties and antimicrobial activity of the smear surface cheese coryneform bacterium *Brevibacterium linens*. Eur Food Res Technol 227:1299–1306. doi:10.1007/s00217-008-0856-4.

[2] Rattray FP, Fox PF 1999 Aspects of enzymology and biochemical properties of *Brevibacterium linens* relevant to cheese ripening: a review. J Dairy Sci 82:891–909. doi:10.3168/jds.S0022-0302(99)75308-7. 10342227

[3] Breed RS 1957 Family IX. Brevibacteriaceae breed 1953, p 490–505. *In* Breed RS, Murray EGD, Smith NR (ed), Bergey’s manual of determinative bacteriology, 7th ed, Williams and Wilkins Co, Baltimore, MD.

[4] Onraedt A, Soetaert W, Vandamme E 2005 Industrial importance of the genus *Brevibacterium*. Biotechnol Lett 27:527–533. doi:10.1007/s10529-005-2878-3. 15973485

[5] Arfi K, Landaud S, Bonnarme P 2006 Evidence for distinct l-methionine catabolic pathways in the yeast *Geotrichum candidum* and the bacterium *Brevibacterium linens*. Appl Environ Microbiol 72:2155–2162. doi:10.1128/AEM.72.3.2155-2162.2006. 16517666 PMC1393222

[6] Bonnarme P, Psoni L, Spinnler HE 2000 Diversity of l-methionine catabolism pathways in cheese-ripening bacteria. Appl Environ Microbiol 66:5514–5517. doi:10.1128/AEM.66.12.5514-5517.2000. 11097940 PMC92494

[7] Kim SH, Park YH, Schmidt-Dannert C, Lee PC 2010 Redesign, reconstruction, and directed extension of the *Brevibacterium linens* C_40_ carotenoid pathway in *Escherichia coli*. Appl Environ Microbiol 76:5199–5206. doi:10.1128/AEM.00263-10. 20525861 PMC2916479

[8] Chin CS, Alexander DH, Marks P, Klammer AA, Drake J, Heiner C, Clum A, Copeland A, Huddleston J, Eichler EE, Turner SW, Korlach J 2013 Nonhybrid, finished microbial genome assemblies from long-read SMRT sequencing data. Nat Methods 10:563–569. doi:10.1038/nmeth.2474. 23644548

[9] Chaisson MJ, Tesler G 2012 Mapping single molecule sequencing reads using basic local alignment with successive refinement (BLASR): application and theory. BMC Bioinformatics 13:238. doi:10.1186/1471-2105-13-238. 22988817 PMC3572422

[10] Myers EW, Sutton GG, Delcher AL, Dew IM, Fasulo DP, Flanigan MJ, Kravitz SA, Mobarry CM, Reinert KH, Remington KA, Anson EL, Bolanos RA, Chou HH, Jordan CM, Halpern AL, Lonardi S, Beasley EM, Brandon RC, Chen L, Dunn PJ, Lai Z, Liang Y, Nusskern DR, Zhan M, Zhang Q, Zheng X, Rubin GM, Adams MD, Venter JC 2000 A whole-genome assembly of *Drosophila*. Science 287:2196–2204. doi:10.1126/science.287.5461.2196. 10731133

[11] Overbeek R, Olson R, Pusch GD, Olsen GJ, Davis JJ, Disz T, Edwards RA, Gerdes S, Parrello B, Shukla M, Vonstein V, Wattam AR, Xia F, Stevens R 2014 The SEED and the rapid annotation of microbial genomes using subsystems technology (RAST). Nucleic Acids Res 42:D206–D214. doi:10.1093/nar/gkt1226. 24293654 PMC3965101

[12] Altschul SF, Madden TL, Schäffer AA, Zhang J, Zhang Z, Miller W, Lipman DJ 1997 Gapped BLAST and PSI-BLAST: a new generation of protein database search programs. Nucleic Acids Res 25:3389–3402. doi:10.1093/nar/25.17.3389. 9254694 PMC146917

[13] van Heel AJ, de Jong A, Montalbán-López M, Kok J, Kuipers OP 2013 BAGEL3: automated identification of genes encoding bacteriocins and (non-) bactericidal posttranslationally modified peptides. Nucleic Acids Res 41:W448–W453. doi:10.1093/nar/gkt391. 23677608 PMC3692055

[14] Joensen KG, Scheutz F, Lund O, Hasman H, Kaas RS, Nielsen EM, Aarestrup FM 2014 Real-time whole-genome sequencing for routine typing, surveillance, and outbreak detection of verotoxigenic *Escherichia coli*. J Clin Microbiol 52:1501–1510. doi:10.1128/JCM.03617-13. 24574290 PMC3993690

[15] Chen L, Xiong Z, Sun L, Yang J, Jin Q 2012 VFDB 2012 update: toward the genetic diversity and molecular evolution of bacterial virulence factors. Nucleic Acids Res 40:D641–D645. doi:10.1093/nar/gkr989. 22067448 PMC3245122

[16] Chakraborty A, Ghosh S, Chowdhary G, Maulik U, Chakrabarti S 2012 DBETH: a database of bacterial exotoxins for human. Nucleic Acids Res 40:D615–D620. doi:10.1093/nar/gkr942. 22102573 PMC3244994

[17] Arndt D, Grant JR, Marcu A, Sajed T, Pon A, Liang Y, Wishart DS 2016 PHASTER: a better, faster version of the PHAST phage search tool. Nucleic Acids Res 44:W16–W21. doi:10.1093/nar/gkw387. 27141966 PMC4987931

[18] Roberts RJ, Carneiro MO, Schatz MC 2013 The advantages of SMRT sequencing. Genome Biol 14:405. doi:10.1186/gb-2013-14-6-405. 23822731 PMC3953343

[19] Roberts RJ, Vincze T, Posfai J, Macelis D 2015 REBASE—a database for DNA restriction and modification: enzymes, genes and genomes. Nucleic Acids Res 43:D298–D299. doi:10.1093/nar/gku1046. 25378308 PMC4383893

[20] Murray IA, Clark TA, Morgan RD, Boitano M, Anton BP, Luong K, Fomenkov A, Turner SW, Korlach J, Roberts RJ 2012 The methylomes of six bacteria. Nucleic Acids Res 40:11450–11462. doi:10.1093/nar/gks891. 23034806 PMC3526280

